# Polish Nurses’ Opinions on the Expansion of Their Competences—Cross-Sectional Study

**DOI:** 10.3390/nursrep11020029

**Published:** 2021-04-29

**Authors:** Kinga Harpula, Anna Bartosiewicz, Jerzy Krukowski

**Affiliations:** 1 Medical College, University of Information Technology and Management in Rzeszów, 35-225 Rzeszów, Poland; wilkinga@gmail.com; 2 Healthcare Complex No. 2, Specialist Outpatient Clinic, Diagnostic Center, 35-005 Rzeszów, Poland; 3 Institute of Health Sciences, Medical College of Rzeszów University, 35-959 Rzeszów, Poland; 4 Faculty of Health Sciences with the Institute of Maritime and Tropical Medicine, Institute of Nursing and Midwifery, Medical University of Gdańsk, 80-211 Gdańsk, Poland; jrkrukowski@gmail.com; 5 Palium Foundation, 89-600 Chojnice, Poland

**Keywords:** nurse, new competences, nursing advice, professional development, advanced practice nurse

## Abstract

The development of medical science creates new challenges for nurses to acquire new skills. Thanks to legal changes in Poland, nurses have gained the opportunity to independently provide health services in many areas, including consultations for patients. The aim of the survey is to analyze nurses’ opinions on the expansion of competences in their profession. This is a cross-sectional, descriptive study conducted among 798 nurses using the survey technique. The majority (65.48%) of the respondents believed that they were adequately prepared to take up new competences. Most of the respondents believed that the new competence would improve the efficiency of the healthcare system in Poland (71.06%) and facilitate patients’ access to health services (65.29%). According to the nurses, the scope of nursing advice will mainly concern the promotion of health education, wound treatment and prescribing medications. Age, seniority and education level significantly influenced the nurses’ opinions on the scope of nursing advice. The Mann–Whitney test and the Kruskal–Wallis test were used. A correlation between two quantitative variables was assessed with the Spearman’s rho coefficient. The significance level of *p* < 0.05 was assumed. The extension of the professional competences of nurses will increase the prestige of the profession and is another step toward introducing the role of Advanced Practice Nurse in Poland.

## 1. Introduction

For many years, the right to consult patients, prescribe medications and perform highly specialized procedures were reserved for the medical profession [1,2,3]. This has changed, with an increasing number of countries worldwide having introduced legal regulations extending the competences of nurses. The United States (USA) and Canada have a long tradition of nurses taking advanced roles, including prescribing medications and counseling patients, and working as equal members of the multidisciplinary team caring for the patient [2,4,5,6]. The factors responsible for the above changes are often the shortage of doctors [7,8] and an increase in the level of nursing education at universities [9,10,11,12]. From January 2016, nurses in Poland obtained the right to prescribe prescriptions, which improved the standard of patient care and access to medical services whilst also improving the professional status of nurses [13]. In January 2020, a new competence for nurses working in Outpatient Specialist Care entered the system, which, in accordance with the regulations, can be implemented in four areas: general surgery, cardiology and diabetology in the case of nurses, and gynecology and obstetrics in the case of midwives [14], while from 1st August 2020, the scope of nursing advice has been extended to Primary Health Care nurses [15]. Nursing advice is to serve those patients who do not require immediate medical intervention, but need a visit to help with a referral, prescription or ongoing health checks. According to the ordinance of the Health Minister, the competences of nurses will be extended to specific cases and in no case are they intended to impose excessive obligations on nurses or undermine the role of doctors in the treatment process [15]. Similar regulations function successfully in other countries, including Great Britain, Finland, Ireland, USA, Australia and Canada [4,16]. In many countries, empowering nurses to prescribe medication is synonymous with the ability to perform a physical examination, write test referrals and consult patients. In Poland, nursing advice is a separate entitlement which, in combination with the previous entitlement to prescribe, constitutes a high level of professional entitlement. The new qualifications are granted to nurses who hold a bachelor’s or master’s degree or hold the title of specialist in nursing [14,15].

New competences give qualified nurses greater independence and will relieve family doctors from some of their duties, helping them to carry out examinations or refer patients to specialist doctors. This is a breakthrough in Polish nursing and another step bringing us closer to other countries, where a nurse as an equal member of the interdisciplinary team together with a doctor provides professional patient care [16].

The aim of the study was to analyze the opinions of nurses on the extension of competences allowing nurses in Poland to provide advice to patients.

## 2. Materials and Methods

### 2.1. Participants and Study Design

The cross-sectional descriptive study was conducted in 2020 among nurses working in various medical entitles (hospitals, primary healthcare, outpatient specialist care, long-term care, health resorts, hospices, psychiatric care and the private sector of healthcare) in the Subcarpathian region of Poland (South-Eastern Poland). Invitations to participate in the study were sent to medical entities, randomly selected via a randomized algorithm program. The sample size was determined with the help of the EPI INFO (StatCalc) software. A multistage random cluster sampling method was used. The message contained data on the planned research. The following criteria for the selection of respondents were used: professionally active nurses, willing to participate in the study. All nurses who consented to participate in the study were thoroughly informed in writing and verbally about the details and purpose of the study, as well as assured of anonymity and voluntary participation, with the possibility of opting out at any stage without any consequences. The survey questionnaires, together with the consent, were delivered to the medical entities that responded positively to the invitation. In order to ensure the confidentiality of data, the questionnaires were numbered, and the completed questionnaires were returned in sealed envelopes previously attached to the questionnaire. A correctly completed questionnaire automatically constituted consent to the participation of a given nurse in the study. Ultimately, 1500 questionnaires were distributed, 812 were collected back and 24 were rejected due to incomplete responses. Data from 798 questionnaires were put on an encoded excel sheet and subjected to statistical analysis. The nurses included in the study were a representative group of all nurses working in the region (the error threshold was 3%, i.e., the test power was 0.97).

### 2.2. Questionnaire

The method used was a diagnostic survey using the survey technique. The first part of the questionnaire included questions concerning the views of nurses on the new competences and the scope of nursing advice. The respondents answered using the five-point Likert scale: from definitely yes to definitely not. Part two contained socio-demographic data of the respondents (age, sex, seniority, place of work, level of education, additional qualifications).

### 2.3. Statistical Analysis

The estimation method and the following statistical methods were used: in order to present the data, the method of descriptive statistics was used—arithmetic mean (M), the value of which determines the average level of a given variable, and standard deviation (SD), a statistical measure of scattering the results around the expected value. The Mann–Whitney test and the Kruskal–Wallis and Spearman’s rho coefficient tests were used.

A significance level of *p* < 0.05 was assumed.

Calculations were performed with the IBM SPSS program Statistics 20 (IMB, Armonk, NY, USA).

### 2.4. Ethics

The study was approved by the institutional Bioethics Committee of the Rzeszow University (Resolution No. 10/12/2020) and all relevant administrative bodies.

## 3. Results

### 3.1. Characteristics of the Studied Group

The study included a group of 798 nurses. The mean age of the respondents was 39.65 years (SD = 11.73) and ranged from 21 to 70 years. The average work experience in the profession was 16.42 years (SD = 11.99). The majority of the studied group were women (*N* = 776, i.e., 97.24%). Most nurses had bachelor level education (38.97%), 36.47% of nurses had a master’s degree, and the least numerous groups included nurses with secondary medical education—24.56%. The most frequently surveyed nurses performed their work as part of a hospital’s services (40.73%) or primary healthcare (35.84%). Additional qualifications possessed by the respondents included, first of all, a completed specialist course (62.28%). Less than half of the respondents had a qualification course in the field of nursing (47.99%), a training course (35.46%) and specialization in the field of nursing (30.70%). 6.52% of nurses had other forms of professional development, while 3.88% of the nurses did not have any additional education (Table 1).

### 3.2. Results

**Figure 1:**
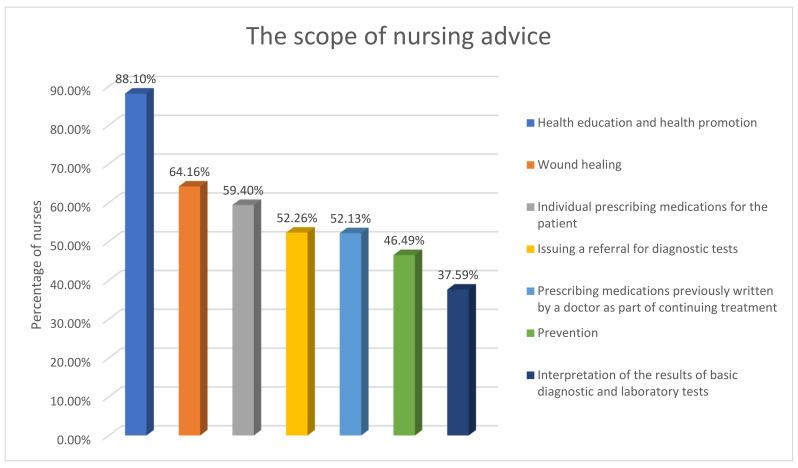
Nurses’ opinion on the scope of nursing advice.

The nurses argued that the scope of “nursing advice” should most often include health promotion and health education (88.10%). More than half of the people stated that the “nursing advice” should include the selection of a wound treatment method (64.16%), prescribing certain medicinal products and medical devices to which the nurse is entitled (59.40%), issuing referrals for basic diagnostic tests, including laboratory tests (52.26%), or prescribing medicinal products prescribed by a doctor as a follow-up (52.13%). As much as 46.49% of the respondents indicated that the “nursing advice” should include the interpretation of the results of basic diagnostic tests, including the results of laboratory tests (Figure 1).

The results of our research showed that the majority of respondents (71.06%) agreed with the opinion that, thanks to the extension of nursing competences and the possibility of providing “nursing advice”, the functioning of the healthcare system in Poland can be improved. More than half of the respondents agreed with the statement that “nursing advice” can facilitate patients’ access to health services (65.79%), that without the help of a doctor, they are able to properly consult the patient (73.29%), that they have the appropriate competences to do it, considered that it will increase the prestige of the nursing profession (74.31%) (Table 2).

Socio-demographic variables such as age, seniority and education level influenced nurses’ opinion on the scope of nursing advice. The age of the respondents had a significant impact on all scopes of nursing advice, except for the interpretation of the results of basic diagnostic and laboratory tests. Work experience had a statistically significant impact on prescribing medications for the patients, issuing a referral for diagnostic tests and prevention. Education was a factor that statistically influenced all scopes of nursing advice—the higher the education, the more often the given scope of nursing advice was indicated. The assessment of nurses’ self-preparation was statistically significant in all areas of nursing advice, except for health education and health promotion (Table 3, Table 4, Table 5 and Table 6).

## 4. Discussion

To our knowledge, this study is the first in Poland to examine the opinions of nurses about the upcoming extension of nurses’ competences related to providing advice to patients. Although in Poland these regulations are novel and sometimes raise many doubts [17], for many years they have been successfully functioning in healthcare systems in other countries, such as Finland, Ireland, Great Britain, USA, Canada and Australia [2,8,18,19]. According to the surveyed nurses, new competences increase the effectiveness of the healthcare system, improve patients’ access to medical services and increase the prestige of the profession. Many studies of Advanced Practice Nursing indicate benefits for the healthcare system, for patients and nurses themselves, taking into account both the economic and quality aspects of the provided solutions [1,2,3,4,19,20]. Therefore, nursing consultations have been recognized as an effective and efficient tool in healthcare [19,20].

International studies in this area emphasize the high level of nurses’ competences and recommend their application in particular to primary healthcare entities. The international debate on the resources of medical personnel clearly shows the relationship between the efficient functioning of the healthcare systems and the skillful delegation of nurses’ potential, especially when there is a shortage of doctors [21,22,23].

The predicted demographic changes and a larger number of people with chronic diseases will trigger further demands for medical services, and taking strategic actions and supporting or substituting doctors with nurses will be one of the important solutions to improve the availability, efficiency and quality of medical care in a given country [22].

The results of the studies conducted in 2015–2017 confirm the legitimacy of remodeling the interdisciplinary cooperation in primary care, pointing to numerous benefits, such as more prescriptions for patients, more referrals for diagnostic tests, the continuity of care without the need to wait for a medical visit and the higher availability of nurses compared to doctors. The demonstrated benefits also include a reduction in the number of deaths in groups of patients with chronic diseases and a higher level of patient satisfaction with the care provided [22,23,24].

The opinions of Polish nurses obtained in this study confirm the above considerations. The draft ordinance assumed that nursing advice would replace medical advice from 3% to 7% out of all the advice currently provided by doctors. [17]. A Cochrane report based on a systematic review of studies in the Netherlands (two studies), Ireland (one study), the United States (eight studies) and the United Kingdom (three studies) shows that nurses were as effective in prescribing medications as physicians for a range of conditions including chronic diseases [4,24,25].

The results showed that, in the opinion of the surveyed nurses, nursing advice will mostly concern health promotion and health education, then wound treatment and the self-prescription of medicinal products and medical devices, issuing referrals for diagnostic tests and prescribing medications as part of a continuing treatment prescribed by a doctor. The scope of the services provided by nurses in individual countries varies, depending on the quality of the healthcare system, legal regulations and the prestige of the nursing profession in a given country [2,4,5,6]. In the USA, nurses are an important link in healthcare and provide a wide range of medical services in the field of patient assessment, ordering, performing and interpreting diagnostic tests, making diagnosis, managing the therapeutic process (managing treatment, including prescribing medication and non-pharmacologic treatments, patient care coordination, as well as counseling and health education [1,2,3,4,25,26,27]). The same holds for Canada, where, apart from the services indicated above, nurses perform minor surgical procedures, e.g., set fractures and admit and discharge patients from hospital [2]. Our results showed that age, seniority and level of education were factors that influenced the nurses’ opinion regarding new competences. Numminen et al. confirm these dependencies [28]. In contrast, Gutiérrez-Rodríguez et al. indicate that a master’s degree or even a doctorate is not always a factor that significantly influences the level of competence [29].

The study has some limitations resulting from the essence of the cross-sectional study, as well as those related to the territorial limitation. Therefore, subsequent studies should have a nationwide scope. Due to the fact that the study was conducted shortly after the introduction of the above-mentioned regulations, the assessment of its quality and effectiveness will be possible only in the coming years.

## 5. Conclusions

Nurses in Poland believe that they are prepared to give advice to patients. In their opinion, this will improve the functioning of the healthcare system, increasing patients’ access to health services and strengthening the prestige of the nursing profession.

## Figures and Tables

**Table 1:** Characteristics of the surveyed nurses (*N* = 798).

Independent Variables	Categories	*N*	%
Age	up to 30 years	238	29.82
	from 31–40 years old	146	18.3
	from 41–50 years old	270	33.83
	over 50 years	144	18.05
Sex	male	22	2.76
	female	776	97.24
Work experience as a nurse	from 1–5 years	223	27.94
	from 6–15 years	161	20.18
	from 16–25 years	211	26.44
	over 25 years	203	25.44
Education	secondary nursing education	196	24.56
	bachelor	311	38.97
	master degree	291	36.47
* Additional qualifications	specialist course	497	62.32
	qualification course	383	47.99
	training course	283	35.46
	specialization	245	30.7
	other forms of professional development	52	6.52
	no additional qualifications	31	3.88
* Place of work	Hospital	325	40.73
	Primary Healthcare	286	35.84
	Long-term-care	92	11.53
	Outpatient Specialist Care (OSC)	84	10.65
	Health resorts	37	4.64
	Hospices	36	4.51
	Private sector	16	2.13
	Psychiatric care	12	1.50

* The values do not add up to 100 as some nurses had more than one additional qualification or were employed in more than one place of work.

**Table 2:** Nurses’ opinion on nursing advice and self-preparation to take on new competences.

Opinion on Nursing Advice According to the Surveyed Nurses	Definitely Yes (%)	Yes (%)	I Have No Opinion (%)	No (%)	Definitely Not (%)
The new competence improves the healthcare system	24.69	46.37	13.41	13.53	2.01
The new competence increases patients’ access to health services	19.80	45.99	14.04	19.05	19.80
I do not need to consult with a doctor to properly consult the patient	12.66	50.63	13.53	15.91	7.20
I am competent to properly perform a consultation for a patient	13.16	52.38	16.29	17.17	1.0
The new competences strengthen the prestige of the nursing profession	34.71	40.60	11.78	10.40	2.51

**Table 3:** The scope of nursing advice depending on the age of the surveyed nurses.

The Scope of Nursing Advice		Age					*p*
		Average	SD	Min.	Maks.	Me	
Prescribing medications for the patient	No	40.94	11.51	21	70	41	0.0128
	Yes	38.78	11.82	21	68	42	
Prescribing medications previously prescribed by a doctor as part of a continuing treatment	No	41.28	11.06	21	70	42	0.0002
	Yes	38.16	12.14	21	68	40	
Issuing a referral for diagnostic tests	No	41.07	11.50	21	70	42	0.0012
	Yes	38.36	11.81	21	66	41	
Interpretation of the results of basic diagnostic and laboratory tests	No	39.91	11.67	21	70	41	0.4857
	Yes	39.23	11.85	21	66	42	
Wound healing	No	40.77	12.23	21	68	42	0.0495
	Yes	39.03	11.41	21	70	41	
Health education and health promotion	No	42.21	11.64	22	66	44	0.0288
	Yes	39.31	11.71	21	70	41	
Prevention	No	40.85	11.69	21	68	42	0.0026
	Yes	38.27	11.65	21	70	40	

**Table 4:** The scope of nursing advice depending on the work experience of the surveyed nurses.

The Scope of Nursing Advice		Work Experience (In Year)					*p*
		Average	SD	Min.	Maks.	Me	
Prescribing medications for the patient	No	1752	12.04	0	45	17	0.0313
	Yes	15.66	11.91	0	44	16	
Prescribing medications previously prescribed by a doctor as part of a continuing treatment	No	18.12	11.57	0	45	18	0.0000
	Yes	14.86	12.17	0	45	15	
Issuing a referral for diagnostic tests	No	17.73	12.12	0	45	18	0.0029
	Yes	15.22	11.75	0	40	15	
Interpretation of the results of basic diagnostic and laboratory tests	No	16.69	12.02	0	45	17	0.4442
	Yes	15.97	11.94	0	40	17	
Wound healing	No	17.45	12.45	0	45	18	0.0931
	Yes	15.84	11.70	0	45	16	
Health education and health promotion	No	18.37	11.96	0	45	19	0.0936
	Yes	16.15	11.98	0	45	16	
Prevention	No	17.70	12.14	0	45	18	0.0011
	Yes	14.94	11.66	0	45	15	

**Table 5:** The scope of nursing advice depending on the level of education of the surveyed nurses.

The Scope of Nursing Advice		Education of Level						*p*
		Secondary Nursing Education		Bachelor’s Degree		Master Degree		
		*N*	%	*N*	%	*N*	%	
Prescribing medications for the patient	No	107	54.6	124	39.9	93	32.0	χ2 = 24.989; *p* = 0
	Yes	89	45.4	187	60.1	198	68.0	
Prescribing medications previously prescribed by a doctor as part of a continuing treatment	No	131	66.8	126	40.5	125	43.0	χ2 = 37.814; *p* = 0
	Yes	65	33.2	185	59.5	166	57.0	
Issuing a referral for diagnostic tests	No	132	67.3	135	43.4	114	39.2	χ2 = 41.096; *p* = 0
	Yes	64	32.7	176	56.6	177	60.8	
Interpretation of the results of basic diagnostic and laboratory tests	No	146	74.5	190	61.1	162	55.7	χ2 = 18.055; *p* = 0.0001
	Yes	50	25.5	121	38.9	129	44.3	
Wound healing	No	90	45.9	118	37.9	78	26.8	χ2 = 19.588; *p* = 0.0001
	Yes	106	54.1	193	62.1	213	73.2	
Health education and health promotion	No	28	14.3	36	11.6	31	10.7	χ2 = 1.526; *p* = 0.4662
	Yes	168	85.7	275	88.4	260	89.3	
Prevention	No	135	68.9	167	53.7	125	43.0	χ2 = 31.642; *p* = 0
	Yes	61	31.1	144	46.3	166	57.0	

**Table 6:** Self-assessment of own competences and the preferred scope of nursing advice.

The Scope of Nursing Advice		I am Competent to Consult a Patient					*p*
		Average	SD	Min.	Maks.	Me	
Prescribing medications for the patient	No	2.61	0.97	1	5	2	0.0000
	Yes	2.26	0.91	1	5	2	
Prescribing medications previously prescribed by a doctor as part of a continuing treatment	No	2.49	0.94	1	5	2	0.0063
	Yes	2.32	0.96	1	5	2	
Issuing a referral for diagnostic tests	No	2.59	0.97	1	5	2	0.0000
	Yes	2.24	0.91	1	4	2	
Interpretation of the results of basic diagnostic and laboratory tests	No	2.55	0.95	1	5	2	0.0000
	Yes	2.17	0.92	1	4	2	
Wound healing	No	2.65	1.00	1	5	2	0.0000
	Yes	2.27	0.90	1	4	2	
Health education and health promotion	No	2.43	1.04	1	5	2	0.7912
	Yes	2.40	0.94	1	5	2	
Prevention	No	2.49	0.97	1	5	2	0.0030
	Yes	2.30	0.92	1	5	2	

## Data Availability

The data presented in this study are available on reasonable request from the corresponding author.

## References

[1] Maier C.B., Aiken L.H., Busse R. (2017). Nurses in advanced roles: Policy levers to implementation. OECD Health Work. Paper.

[2] Kroezen M., Francke A.L., Groenewegen P.P., van Dijk L. (2012). Nurse prescribing of medicines in Western European and Anglo-Saxon countries: A survey on forces, conditions and jurisdictional control. Int. J. Nurs. Stud..

[3] Maier C.B., Aiken L.H. (2016). Task shifting from physicians to nurses in primary care in 39 countries: A cross-country comparative study. Eur. J. Public Health.

[4] Weeks G., George J., Maclure K., Steward D. (2016). Non-medical prescribing versus medical prescribing for acute and chronic disease management in primary and secondary care. Cochrane Database Syst Rev..

[5] Gielen S.C., Dekker J., Francke A.L., Mistiaen P., Kroezen M. (2014). The effects of nurse prescribing: A systematic review. Int. J. Nurs. Stud..

[6] Ross J. (2012). Nurse prescibing in the USA: A nurse prescribing practice report. Nurse Prescr..

[7] Health at a Glance: Europe 2018. State of health in the EU Cycle. https://read.oecd-ilibrary.org/social-issues-migration-health/health-at-a-glance-europe-2018_health_glance_eur-2018-en#page180.

[8] Bodenheimer T.S., Smith M.D. (2013). Primary care: Proposed solutions to the physician shortage without training more physicians. Health Aff..

[9] Felton A., Royal J. (2015). Skills for nursing practice: Development of clinical skills in pre-registration nurse education. Nurse Educ. Pract..

[10] Haddad M., Butler G.S., Tylee A. (2010). School nurses’ involvement, attitudes and training needs for mental health work: A UK wide cross sectional study. J. Adv. Nurs..

[11] Cashin A., Heartfield M., Bryce J., Devey L., Buckley T., Cox D., Fisher M. (2017). Standards for practice for registered nurses in Australia. Collegian.

[12] Leigh J., Roberts D. (2018). Critical exploration of the new NMC standards of proficiency for registered nurses. Br. J. Nurs..

[13] Act of 22 July 2014 Amending the Act on the Profession of Nurse and Midwife and Some Other Acts (Journal of Laws of 2014, Item 1136). http://isap.sejm.gov.pl/isap.nsf/download.xsp/WDU20140001136/O/D20141136.pdf..

[14] Regulation of the Minister of Health of 23 September 2019 Amending the Regulation on Guaranteed Services in the Field of Outpatient Specialist Care. Journal of Laws 2019, Item 1864. http://isap.sejm.gov.pl/isap.nsf/download.xsp/WDU20190001864/O/D20191864.pdf.

[15] Regulation of the Minister of Health of 8 July 2020 Amending the Regulation on Guaranteed Benefits in the Field of Primary Health care. Journal of Laws 2020, Item 1225. https://isap.sejm.gov.pl/isap.nsf/download.xsp/WDU20200001255/O/D20201255.pdf.

[16] Maier C.B. (2019). Nurse prescribing of medicines in 13 European countries. Hum. Resour. Health.

[17] Project of Regulation of the Health Minister Amending the Ordinance on Guaranteed Services in the Field of Primary Health care—Regulation Impact Assessment. https://legislacja.rcl.gov.pl/projekt/12330600/katalog/12665158#12665158.

[18] Legge A. (1997). Nurse prescribing is a success. BMJ.

[19] Wilson D.M., Murphy J., Nam M.A., Fahy A., Tella S. (2018). Nurse and midwifery prescribing in Ireland: A scope-of-practice development for worldwide consideration. Nurs. Health Sci..

[20] Venning P., Durie A., Roland M., Roberts C., Leese B. (2000). Randomised controlled trial comparing cost effectiveness of general practitioners and nurse practitioners in primary care. BMJ.

[21] Fagerström L., Glasberg A.L. (2011). The first evaluation of the advanced practice nurse role in Finland–the perspective of nurse leaders. J. Nurs. Manag..

[22] Laurant M., van der Biezen M., Wijers N., Watananirun K., Kontopantelis E., van Vught A.J. (2018). Nurses as substitutes for doctors in primary care. Cochrane Database Syst. Rev..

[23] Heale R., Rieck Buckley C. (2015). An international perspective of advanced practice nursing regulation. Int. Nurs. Rev..

[24] Aiken L.H., Sloane D., Griffiths P., Rafferty A.M., Bruyneel L., McHugh M., Maier C.B., Moreno-Casbas T., Ball J.E., Ausserhofer D. (2017). Nursing skill mix in European hospitals: Cross-sectional study of the association with mortality, patient ratings, and quality of care. BMJ.

[25] Schober M. (2018). Global emergence of nurse practitioner/advanced practice nursing roles. J. Am. Assoc. Nurse Pract..

[26] Rolfe G. (2014). Understanding advanced nursing practice. Nurs. Times.

[27] Perkin K. (2011). Nurse practitioners and interprofessional collaboration. J. Interprof. Care.

[28] Numminen O., Meretoja R., Isoaho H., Leino-Kilpi H. (2013). Professional competence of practising nurses. J. Clin. Nurs..

[29] Gutiérrez-Rodríguez L., Mayor S.G., Lozano D.C., Burgos-Fuentes E., Rodríguez-Gómez S., Sastre-Fullana P., Morales-Asencio J.M. (2019). Competences of specialist nurses and advanced practice nurses. Enfermería Clínica.

